# RETRACTED: Maroni et al. The Autophagic Process Occurs in Human Bone Metastasis and Implicates Molecular Mechanisms Differently Affected by Rab5a in the Early and Late Stages. *Int. J. Mol. Sci.* 2016, *17*, 443

**DOI:** 10.3390/ijms27146508

**Published:** 2026-07-22

**Authors:** Paola Maroni, Paola Bendinelli, Massimo Resnati, Emanuela Matteucci, Enrico Milan, Maria Alfonsina Desiderio

**Affiliations:** 1 Istituto Ortopedico Galeazzi, Scientific Institute for Research, Hospitalization and Health Care (IRCCS), 20161 Milano, Italy; 2 Dipartimento di Scienze Biomediche per la Salute, Molecular Pathology Laboratory, Università degli Studi di Milano, 20133 Milano, Italy; 3 San Raffaele Scientific Institute, Division of Genetics and Cell Biology, 20133 Milano, Italy; resnati.massimo@hsr.it (M.R.);

The journal retracts the article “The Autophagic Process Occurs in Human Bone Metastasis and Implicates Molecular Mechanisms Differently Affected by Rab5a in the Early and Late Stages” [1] cited above.

Following publication, concerns were brought to the attention of the Editorial Office regarding the integrity of the figures presented in this article [1].

In accordance with standard journal procedure, the Editorial Office and Editorial Board conducted an investigation that confirmed the duplication between elements of Figure 4 in this article [1] and Figure 7 presented in a different publication [2], produced by a similar authorship group, presenting different experimental conditions. The Editorial Board has lost confidence in the reliability of the findings and decided to retract this publication [1], as per MDPI’s retraction policy.

This retraction was approved by the Editor-in-Chief of the *International Journal of Molecular Sciences*.

Massimo Resnati and Enrico Milan have been informed of this retraction and have indicated their agreement with this decision. The remaining authors have not provided comment. The authors’ contributions are described in the original article.
